# Development and Characterization of Modified Gelatin-Based Cling Films with Antimicrobial and Antioxidant Activities and Their Application in the Preservation of Cherry Tomatoes

**DOI:** 10.3390/antiox13040431

**Published:** 2024-04-02

**Authors:** Jianfu Qiao, Linjing Wang, Luxin Wang, Ziyan Li, Yue Huai, Shaoying Zhang, Youwei Yu

**Affiliations:** College of Food Science, Shanxi Normal University, Taiyuan 030000, China; 222420012@sxnu.edu.cn (J.Q.); 2230010117@sxnu.edu.cn (L.W.); 2230010222@sxnu.edu.cn (L.W.); 2230010212@sxnu.edu.cn (Z.L.); 2230010211@sxnu.edu.cn (Y.H.)

**Keywords:** gelatin-based cling films, glycidyltrimethylammonium chloride (GTA), rosmarinic acid (RosA), cherry tomato preservation

## Abstract

The utilization of functional cling films presents a promising approach to alleviate post-harvest spoilage caused by microbial activity, oxidative metabolism, and moisture loss in agricultural products. To overcome the environmental problems of conventional packaging materials, in this study, we developed functional fruit and vegetable cling films based on glycidyltrimethylammonium chloride and rosemarinic acid cross-linked gelatin (RQ-GEL). The results indicate that the prepared RQ-GEL film possesses excellent UV light barrier properties and mechanical performance. RQ-GEL inhibited *S. aureus* and *E. coli* by 93.79% and 92.04%, respectively. DPPH and ABTS free radical scavenging activities were as high as 87.69% and 84.6%. In the cherry tomato preservation experiment, when compared to uncovered samples, the RQ-GEL group had a 29.77% reduction in weight loss and a significant 26.92% reduction in hardness. Meanwhile, the RQ-GEL group delays the decline of fruit total soluble solids and titratable acidity content, and prolongs the preservation period of cherry tomatoes. Hence, RQ-GEL cling film is poised to emerge as a promising packaging material for the post-harvest preservation of agricultural products.

## 1. Introduction

Globally, post-harvest losses of fruits and vegetables due to microbial contamination and improper storage range from 15 to 50% [1]. Cherry tomatoes, the world’s second most important vegetable crop in terms of total production value, are rich in carotenoids and antioxidants such as vitamin C, lycopene, and polyphenols, which have beneficial effects on reducing the incidence of cancer and heart disease [2]. Meanwhile, its distinctive flavor and texture are highly favored by consumers. However, post-harvest losses of cherry tomatoes, attributed to their high moisture content, respiratory activity, microbial contamination, and damage during storage and transportation, reach up to 25–42% annually [3]. 

Currently, the primary packaging materials for preserving fruits and vegetables are petroleum-based synthetic polymers such as polyethylene and polypropylene. However, the widespread application of these materials also entails certain adverse effects, including limited performance in active packaging, challenges in biodegradability, and contributing to environmental pollution [4]. Natural macromolecular polymers, including polysaccharides and proteins, can emerge as primary alternatives to petroleum-based polymers in the future due to advantages such as readily available raw materials, low cost, and biodegradability [5]. Thin films of naturally occurring macromolecular materials can establish multifunctional barriers, inhibiting gas exchange, reducing mechanical damage, retarding moisture evaporation, and effectively maintaining the quality of post-harvest fruits and vegetables. Chen et al. extended the shelf life of cherry tomatoes by employing a composite coating of konjac glucomannan and curdlan [6], while Zhao et al. effectively preserved the post-harvest quality of blueberries using a chitosan/Enoki mushroom foot polysaccharide composite cling film [7]. However, these natural macromolecular materials face limitations in antioxidant and antibacterial capabilities, making it challenging to effectively eliminate free radicals such as O_2_^•−^ generated during the respiration process of fruits and vegetables and prevent microbial contamination. Some researchers enhance the antioxidant and antibacterial activities of natural macromolecular materials by incorporating essential oils, such as ginger essential oil [8] and citrus essential oil [9]. However, these physically incorporated substances exhibit significant aggregation and low stability, making them susceptible to migration onto fruits and vegetables or evaporation during preservation processes [10]. Moreover, essential oils inherently possess distinct odors, inevitably impacting the natural flavors of fruits and vegetables during storage, rendering them challenging to promote and apply widely. 

Meanwhile, we also note that gelatin, as a biopolymer, is derived from the skins and bones of animals such as cattle, sheep, and fish. Due to its wide availability, low cost, biodegradability, and excellent film-forming properties, it has become a focal point for researchers in the development of green preservation materials [11]. RosA, a natural polyphenolic compound containing four phenolic hydroxyl groups in its molecular structure, endows it with excellent antioxidant activity [12]. Meanwhile, cationic groups such as GTA exhibit strong bactericidal activity [13]. However, there have been few reports on the preparation of active preservation materials by crosslinking RosA and GTA into gelatin.

Here, we report for the first time a strategy for preparing cling films with excellent antioxidant and antimicrobial activities by introducing rosmarinic acid and glycidyltrimethylammonium chloride (RQ-GEL) into a gelatin matrix. GTA grafting onto gelatin enhances the cling film’s mechanical properties and antimicrobial activity, effectively inhibiting microbial contamination. RosA, by forming hydrogen bonds with the amino groups in gelatin, improves the cling film’s transparency, enhances UV light barrier properties, and endows it with excellent antioxidant activity [14]. Simultaneously applied in the preservation of cherry tomatoes, the preservation performance of the film was assessed based on the physicochemical properties of tomatoes. This endeavor aims to provide application references for the development of novel eco-friendly packaging materials. 

## 2. Materials and Methods

### 2.1. Materials

Cherry tomatoes (Millennium variety) were purchased from Huilong Farmer’s Market in Jinzhong City. Gelatin (Bloom strength ≥ 250 g, from bovine bones), glycidyltrimethylammonium chloride (GTA, ≥95%), and 2,2′-azino-bis(3-ethylbenzothiazoline-6-sulfonic acid) (ABTS) were obtained from Shanghai Aladdin Bio-Chem Technology Co., Ltd (Shanghai, China). Rosmarinic acid (RosA, 97%) and 1,1-diphenyl-2-picrylhydrazyl (DPPH) were purchased from Shanghai Yuan Ye Biotechnology Co., Ltd (Shanghai, China). 2,4,6-trinitrobenzenesulfonic acid was acquired from Sigma-Aldrich (St. Louis, MO, USA). All other chemical reagents were of analytical grade.

### 2.2. Preparation of Quaternized Gelatin (Q-GEL)

First, 2 g of gelatin powder was dissolved in 100 mL of deionized water, ensuring complete dissolution at 45 °C. Subsequently, 1.2 g of GTA was added to the solution, and the mixture was continuously stirred at 55 °C with a speed of 500 rpm for 1.5 h. The reaction liquid was then collected and subjected to freeze-drying. The degree of cross-linking, determined using the TNBS method [15] with absorbance measurements at 345 nm (Background Abs: 0.111, Blank group Abs: 0.558, Experimental group Abs: 0.184), is calculated to be 0.84 (Figure S1).

### 2.3. Preparation of RQ-GEL

The RQ-GEL was prepared according to a reported procedure [14]. Briefly, Q-GEL was initially dissolved in deionized water at 55 °C with continuous stirring for 30 min, resulting in a 2% (*w*/*v*) solution. Subsequently, RosA was added to the Q-GEL solution, and intermittent stirring was conducted for 1 h, achieving a final RosA concentration of 0.8% (*w*/*v*). The resulting film solution (30 mL) was uniformly injected into a 100 × 100 mm polytetrafluoroethylene culture dish and dried for 24 h in an oven at 30 °C. The peeled film was placed in a drying dish containing color-changing silica gel for 48 h as the test sample.

### 2.4. Characterization of RQ-GEL Films 

#### 2.4.1. Transmittance and Opacity

Following the approach outlined by Tan et al. for measuring transmittance and opacity [16], films were cut into 4 × 1 cm strips and affixed to the surface of quartz cuvettes, with blank cuvettes used as controls. The transmittance of the films within the wavelength range of 200–800 nm was measured using a UV-visible spectrophotometer (TU-1901, Puxi General Co., Beijing China). Subsequently, the absorbance values of the films at 600 nm were employed in Equation (1) to calculate the opacity of the films, with six repetitions of the test. The opacity calculation is as follows:

(1) $$\mathrm{Opacity}=\frac{A_{600}}{d}$$

where *A*_600_ is the absorbance value of the film at 600 nm; *d* is the thickness of the film (mm).

#### 2.4.2. Mechanical Properties 

Based on the method reported by Lv et al. [17] with some modifications, the tensile strength (TS) and elongation at break (EAB) were determined using an electronic universal material testing machine (5944, Instron Co., Gardena, CA, USA). Film strips (70 × 10 mm) were cut, with an initial gauge length set to 50 mm and a stretching speed of 50 mm/min. The maximum tension and length at the point of rupture were recorded, with each set repeated 5 times.

TS is calculated as follows:

(2) $$\mathrm{TS}(\mathrm{MPa})=\frac{F_{max}}{\Phi }$$

where *F_max_* is the maximum load (N) needed to pull the sample apart, *Φ* is the cross-sectional area (m^2^) of the samples.

EAB is calculated as follows:

(3) $$\mathrm{EAB}\;(\%)=\frac{L_{1}-L_{0}}{L_{0}}\times 100$$

where *L*_1_ is the length when the film is disconnected and *L*_0_ is the initial length of the film.

#### 2.4.3. Fourier Transform Infrared Spectroscopy (FT-IR) Determination

The interaction between RosA, GTA and gelatin was investigated using Fourier transform infrared spectroscopy (660-IR, Varian, Palo Alto, CA, USA). In all, 1 mg of RQ-GEL and 100 mg of KBr were weighed separately, mixed and ground thoroughly and subsequently compacted into thin tablets using a tablet press. The scan range of the spectra was 500 cm^−1^ to 4000 cm^−1^, with a resolution of 4 cm^−1^ and 32 scans.

#### 2.4.4. X-ray Diffraction (XRD) Determination

The X-ray diffraction (XRD) patterns of the films were obtained using an X-ray diffractometer (Ultima IV-185, Rigaku Co., Tokyo, Japan). The samples were irradiated with Cu-Kα radiation under the conditions of 45 kV and 40 mA and scanned at a rate of 2°/min in the 2θ range of 5–60°.

#### 2.4.5. Thermogravimetric Analysis Determination

Thermogravimetric analysis of films was completed using a thermogravimetric analyzer (TGA/DSC 1/1600 HT, Mettler-Toledo, Zurich, Switzerland). Samples (5 mg) were placed into ceramic crucibles and heated from 30 °C to 600 °C at 10 °C/min under a nitrogen atmosphere.

#### 2.4.6. Scanning Electron Microscope (SEM) Determination

The surface and cross-sectional morphology of the films were observed using a scanning electron microscope (JSM-7500F, JEOL, Tokyo, Japan). The voltage was set to 5 kV, and observations of the samples were conducted at magnifications of 500× (surface) and 1000× (cross-section).

#### 2.4.7. Determination of Antibacterial Activity 

Following the method outlined by Kong et al. for determining pathogenic fungi (*B. cinerea* BNCC123731 and *A. alternata* BNCC115062) [18], Q-GEL and RQ-GEL solutions were dissolved in potato dextrose agar (PDA) medium. The mycelial disks (diameter 10 mm) were divided from the edge of the 7-day-old colony, placed at the center of each PDA culture dish, and incubated at 28 °C for 7 days. The diameter of each colony was measured (mm) and records were captured using a camera.

Adopting the approach outlined by Zhou et al. [10], *S. aureus* CMCC 26003 and *E. coli* ATCC 11229 were diluted to 1 × 10^6^ CFU/mL in PBS solution. Subsequently, Q-GEL and RQ-GEL were individually mixed with bacterial suspensions at a volume ratio of 1:1 and co-cultured for 3 h. Following this, 100 μL of the suspensions were uniformly spread onto the beef paste peptone Petri dishes. Incubation was carried out in a constant temperature incubator at 37 °C for 24 h and colony counting was performed to determine the antibacterial rate. 

#### 2.4.8. Determination of Antioxidant Activity

According to the method of Maryam Moghadam et al. with slight modifications [19], the DPPH free radical scavenging activity was determined. Film pieces of dimensions 20 × 30 mm were placed in 10 mL of deionized water, shaken on a shaker for 12 h, and then centrifuged at 6980 rpm for 10 min. The supernatant (2.0 mL) was mixed with 2 mL of 0.17 mM DPPH ethanol solution, and the mixture was incubated in darkness at room temperature for 30 min. The absorbance of the reaction solution was measured at 517 nm.

(4) $$\mathrm{DPPH}\;\mathrm{scavenging}\;\mathrm{activity}\;(\%)=\frac{A_{0}-A_{1}}{A_{0}}\times 100$$

where *A*_1_ is the absorbance value of the DPPH solution containing the sample and *A*_0_ is the absorbance value of the DPPH solution.

Adapting the approach of Lin et al. with slight modifications [20], the ABTS free radical scavenging capacity was determined. Initially, ABTS solution (7.4 mM) and potassium persulfate solution (2.6 mM) were prepared. Subsequently, 5 mL of ABTS solution was mixed with 5 mL of potassium persulfate solution, and the mixture was left in the dark at room temperature overnight. Before use, the ABTS mixture was further diluted with ethanol to achieve an absorbance of 0.7 at 734 nm. A total of 1 mL of the supernatant was mixed with 2 mL of the ABTS solution, and after standing for 6 min, the absorbance of the solution was measured at 734 nm.

(5) $$\mathrm{ABTS}\;\mathrm{scavenging}\;\mathrm{activity}\;(\%)=\frac{B_{0}-B_{1}}{B_{0}}\times 100$$

where *B*_1_ is the absorbance value of the ABTS solution containing the sample and *B*_0_ is the absorbance value of the ABTS working solution.

### 2.5. Determination of Freshness Preservation

#### 2.5.1. Preservation of Cherry Tomato

Cherry tomatoes of equal size, ripeness, and without mechanical damage were immersed in a 100 mg/L sodium hypochlorite solution for 1 min for disinfection and allowed to air dry naturally. Subsequently, they were placed in plastic storage boxes (110 × 75 mm), with a layer of prepared cling film (130 × 95 mm) covering the top in the experimental group, while the uncovered group served as a control. Finally, the samples were stored at room temperature (18–22 °C, relative humidity 30–50%) for 12 days. Samples were taken on days 0, 3, 6, 9, and 12, and the physicochemical properties of the cherry tomatoes were determined.

#### 2.5.2. Weight Loss and Hardness

The initial mass of the tomatoes is denoted as m_0_, and the mass changes are recorded every 3 days as m_1_, with 3 repeated measurements for each sample group. The mass loss is calculated using the following formula:

(6) $$\mathrm{Weight}\;\mathrm{loss}\;(\%)=\frac{m_{0}-m_{1}}{m_{0}}\times 100$$

According to the reported procedure with slight modifications of Yu et al. [21], cherry tomato hardness was measured using a texture analyzer (TA.XT.plus SMS, Stable Micro Systems, Godalming, UK). The P2 probe was employed to puncture the central region of the tomato at a testing speed of 1 mm/s, with a strain setting of 40%. Each tomato underwent 5 measurements, resulting in a total of 30 measurements for each sample group.

#### 2.5.3. Total Soluble Solids and Titratable Acidity

The total soluble solids content of cherry tomatoes was determined using a digital refractometer (PAL-1, ATAGO Tokyo, Japan). The tomatoes were homogenized thoroughly in a tissue homogenizer (JJ-2, Guohua Electric, Changzhou, China), and a drop of tomato juice was placed on the sample well of the refractometer to measure the total soluble solids, with results expressed as a percentage.

After homogenizing 10 g of tomato samples, the mixture was filtered and transferred to a 100 mL volumetric flask, and diluted to the volume with water. The titration was conducted using a 0.1 mol/L NaOH standard solution with phenolphthalein as the indicator.

### 2.6. Statistical Analysis

Data analysis was conducted using SPSS Statistics 25 software, and the results were presented as mean ± standard deviation. Subsequently, the data were subjected to a one-way analysis of variance (ANOVA) followed by post hoc analysis using Duncan’s multiple range test. Statistical significance was considered at a threshold of *p* < 0.05.

## 3. Results

### 3.1. Transmittance and Opacity

**Figure 1:**
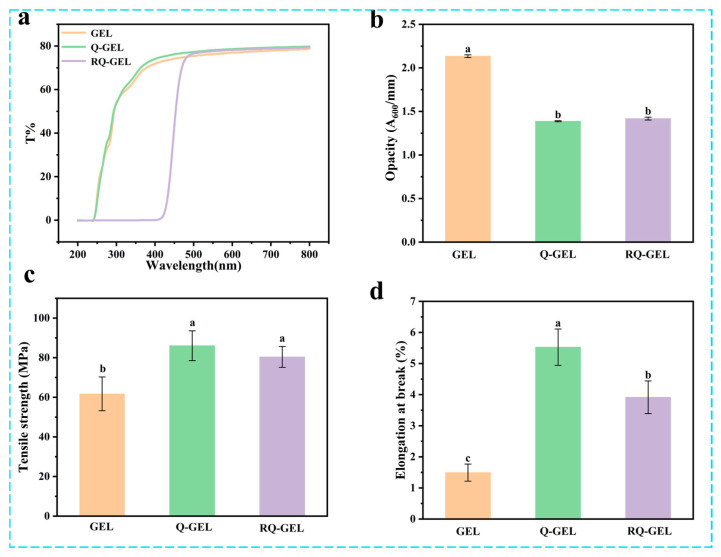
Effect of Q-GEL and RQ-GEL cling film on transmittance (**a**), opacity (**b**), tensile strength (TS) (**c**), and elongation at break (EAB) (**d**). Vertical bars show the standard error of the means and different letters are significantly different at *p* < 0.05.

UV light can induce lipid oxidation in food, and the film’s light barrier property is primarily reflected in its ability to block UV light [22]. The spectral scans of gelatin-based cling films in the UV and visible light range are shown in Figure 1a. Similar to previous studies, the GEL film itself exhibits high transmittance, indicating weak light barrier properties [23]. The transmittance of Q-GEL film is similar to that of GEL film. In the range of 200–416 nm, the transmittance of RQ-GEL film is 0, effectively blocking UV light and demonstrating excellent light barrier performance. This is attributed to the enhanced n-π* absorption in the 200–400 nm range by the phenolic hydroxyl groups introduced by the addition of RosA [14]. Liu et al. observed a significant improvement in UV light absorption when tea polyphenols were added to gelatin [24]. Additionally, it is noteworthy that the RQ-GEL film only reduces transmittance in the range of 200–480 nm. In the visible light range beyond 480 nm, the transmittance of the three films is approximately 75–79%, with no significant differences.

Transparency is a crucial property of preservation films, commonly expressed as opacity. Lower opacity values indicate higher transparency, and vice versa. As depicted in Figure 1b, the opacity value of GEL film is 2.13, significantly higher than that of Q-GEL film (1.39) and RQ-GEL film (1.42). This difference arises from the modification of gelatin with quaternary ammonium salt, and the addition of RosA does not result in an absorption peak at 600 nm. However, the modification of gelatin leads to a substantial increase in thickness (Figure S2). Consequently, the opacity values of Q-GEL and RQ-GEL films decrease, indicating their higher transparency, which is more advantageous for fruit and vegetable packaging applications.

### 3.2. Mechanical Properties

Cling films with robust mechanical properties enhance the resistance of packaged food to external impacts and maintain the integrity of the food. Therefore, cling films need to possess sufficient mechanical strength during transportation, storage, and sales processes. The tensile strength (TS) of gelatin-based cling films is illustrated in Figure 1c. The TS of GEL film is 61.7 MPa, significantly lower than that of Q-GEL film (86.0 MPa) and RQ-GEL film (80.4 MPa). This difference is speculated to be due to the formation of a hydrogen bond and electrostatic interaction network between quaternary ammonium salt groups introduced by gelatin modification and gelatin molecules, resulting in a more compact structure in Q-GEL film. Interestingly, the addition of RosA did not further enhance the TS of RQ-GEL film, contrary to the conclusions of Friesen and Zhang [14,25]. This is because the quaternary ammonium salt cross-linking sites are the -NH_2_ groups on gelatin, and the cross-linking degree of quaternary ammonium salt is as high as 0.84. Only a small fraction of residual -NH_2_ groups can form hydrogen bonds with RosA, and some RosA may not have additional binding sites on the gelatin framework. Additionally, RosA lacks film-forming properties, leading to no significant improvement in the tensile strength of RQ-GEL film compared to Q-GEL film.

The elongation at break (EAB) of Q-GEL and RQ-GEL films is depicted in Figure 1d. The EAB of GEL film is 1.49%, while that of Q-GEL film is 3.7 times higher. This difference may be attributed to the cross-linking reaction between GTA and gelatin, where GTA’s hydrophilic groups form hydrogen bonds with a considerable number of water molecules, resulting in impressive extensibility in Q-GEL film. Notably, the addition of RosA in RQ-GEL film significantly reduces EAB compared to Q-GEL film, suggesting that an excessive amount of RosA is unfavorable for EAB. This observation aligns with the findings of Zhang et al., who investigated the impact of different concentrations of RosA in gelatin films [14].

### 3.3. FT-IR Results and Analysis

**Figure 2:**
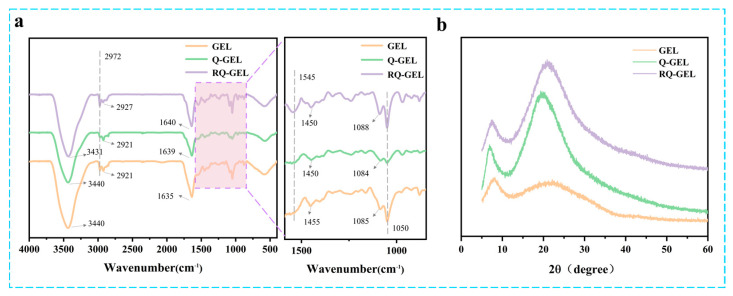
Effect of Q-GEL and RQ-GEL cling film on FT-IR spectroscopy (**a**), XRD (**b**).

Q-GEL and RQ-GEL films exhibit noticeable differences in FT-IR spectra (Figure 2a). In the GEL spectrum, the peak at 1635 cm^−1^ (representing C=O stretching) shifts to 1639 cm^−1^ in the Q-GEL spectrum [22]. The peak at 1455 cm^−1^ (representing N-H bending and C-N stretching) in the Q-GEL spectrum shifts to 1450 cm^−1^ [20], confirming the successful reaction between GTA and gelatin. In the RQ-GEL spectrum, the addition of RosA induces a shift in the amide A band due to N-H stretching and hydrogen bond formation, from 3440 cm^−1^ to 3431 cm^−1^ [26]. This is consistent with Wu et al., who observed a similar shift in the amide A band of gelatin films when adding green tea extract, from 3318 cm^−1^ to 3302 cm^−1^ [27]. In the RQ-GEL spectrum, the peak at 2921 cm^−1^ (representing CH_2_ stretching) shifts to 2927 cm^−1^. A new peak appears at 1545 cm^−1^ (representing C-N stretching and N-H bending). Additionally, the peak at 1084 cm^−1^ in the Q-GEL spectrum shifts to 1088 cm^−1^ in the RQ-GEL spectrum, attributed to the enhanced C-O stretching between gelatin and phenolic compounds [17]. These results indicate the presence of RosA in RQ-GEL.

### 3.4. XRD Results and Analysis

The XRD spectra of Q-GEL and RQ-GEL films are depicted in Figure 2b. All the samples broad peak near 2θ = 20°, indicating that all samples have an amorphous structure. The peak at 2θ = 7° indicates the semicrystalline structure of the RQ-GEL film [17]. The position and intensity of the 2θ = 7° peak are closely related to the size and content of the triple-helix structure of gelatin [28]. Notably, compared to the gelatin film, the Q-GEL film shows a significant enhancement in the intensity of the two diffraction peaks at 2θ = 7° and 2θ = 20°, suggesting a strong interaction force between GTA and gelatin. This observation aligns with the data from the crosslinking degree measurements (Figure S1). The intensity of the two main diffraction peaks of the RQ-GEL film is slightly decreased compared to that of the Q-GEL film, indicating a weak interaction (hydrogen bonding) between RosA and gelatin.

### 3.5. Thermogravimetric Analysis Results and Analysis

**Figure 3:**
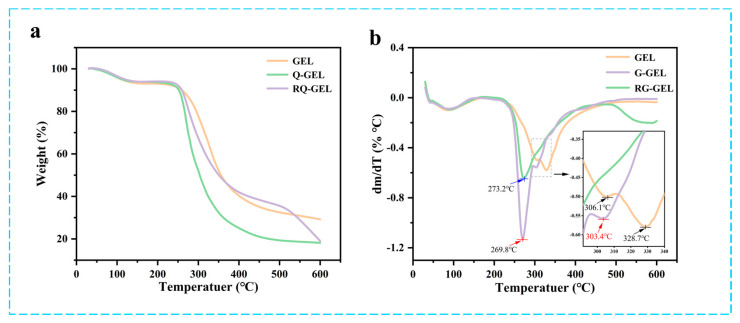
Effect of Q-GEL and RQ-GEL cling film on TGA thermograms (**a**), DTG curves (**b**).

The TGA curves and DTG curves of Q-GEL and RQ-GEL films are presented in Figure 3a,b. The TGA curves of the films exhibit changes with temperature. The first stage occurs in the temperature range of 25–150 °C, primarily involving the loss of moisture in the films (GEL weight loss is approximately 8%, Q-GEL about 7%, RQ-GEL about 7%). The second stage of the films occurs in the range of 250–450 °C (GEL about 57%, Q-GEL about 72%, RQ-GEL about 55%), mainly attributed to protein degradation [22]. Studies by Neo et al. suggest that as the temperature rises, the degradation rate of gelatin gradually accelerates [29]. Interestingly, GEL and Q-GEL do not exhibit a distinct third-stage degradation. However, the RQ-GEL film shows a noticeable third-stage degradation around 500–600 °C (RQ-GEL about 16.4%). This is attributed to the addition of RosA, which forms hydrogen bonds with amino groups on gelatin, leading to rapid degradation when reaching its decomposition temperature in the range of 500–600 °C. The DTG curves indicate downward peaks corresponding to the temperature of maximum weight loss. The maximum weight loss temperatures for GEL, Q-GEL, and RQ-GEL are 328.7 °C, 269.8 °C, and 273.2 °C, respectively. Overall, the thermal stability of Q-GEL and RQ-GEL films remains stable within the temperature range of 200 °C, showcasing potential application potential in conventional food packaging. 

### 3.6. SEM Results and Analysis 

**Figure 4:**
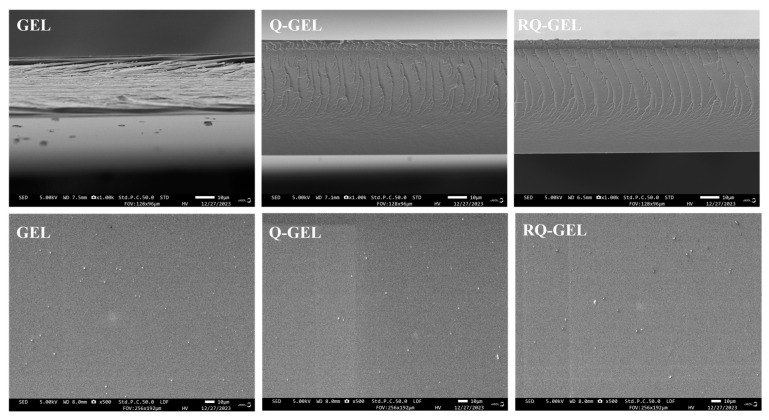
Effect of Q-GEL and RQ-GEL cling film on SEM.

SEM images reveal some changes in the microstructure of the films. The SEM micrographs of the surface and cross-section of Q-GEL and RQ-GEL films are depicted in Figure 4. The surface of the GEL film exhibits slight protrusions, with no apparent cracks or pores, presenting an overall relatively smooth appearance. The modified Q-GEL and RQ-GEL do not compromise the uniformity of the films, and their surface morphology shows no noticeable difference compared to the control film. In cross-section, the GEL film displays a layered structure, and gaps between film cross-sections are evident. In comparison to the GEL film, the thickness of the Q-GEL and RQ-GEL films significantly increases (Figure S2), consistent with the measured thickness indicators. The cross-section of Q-GEL appears dense, while that of RQ-GEL exhibits a fish-scale-like morphology.

### 3.7. Determination of Antibacterial Activity

#### 3.7.1. Fungi

**Figure 5:**
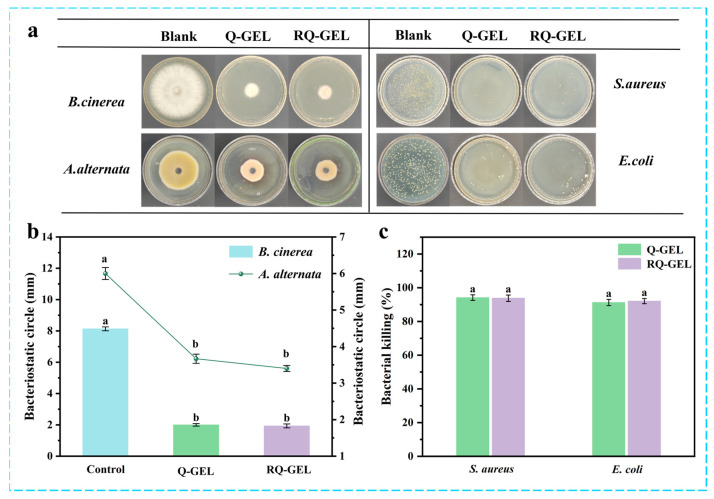
(**a**) Bacteriostatic electronic photographs of Q-GEL and RQ-GEL cling film. Effect of Q-GEL and RQ-GEL cling film on fungal inhibitory circle (**b**), antibacterial rate (**c**). Vertical bars show the standard error of the means and different letters are significantly different at *p* < 0.05.

Agricultural products are susceptible to pathogenic fungal infections during post-harvest transportation due to mechanical damage caused by collisions and weak epidermis [30]. Antimicrobial films can inhibit the growth of these microorganisms, providing a safe barrier for food. Figure 5a presents electron photographs depicting the antibacterial effect of the RQ-GEL, and Figure 5b illustrates the inhibition zones of the RQ-GEL against *B. cinerea* and *A. alternata*. In the blank control group, the observed inhibition zones for *B. cinerea* and *A. alternata* are 81.3 mm and 60.0 mm, respectively, while the inhibition zones for the Q-GEL group are 20.0 mm and 36.7 mm, and for the RQ-GEL group are 19.3 mm and 34.0 mm. The significantly reduced inhibition zones of the Q-GEL and RQ-GEL groups compared to the blank control group indicate their strong antibacterial properties. Xu et al. demonstrated excellent antibacterial effects against *Aspergillusniger* and *Penicilliumcitrinum* using prepared rosin quaternary ammonium salts [31]. Judging from the inhibitory effects in the inhibition zones, both Q-GEL and RQ-GEL groups exhibit stronger suppression against *B. cinerea* compared to *A. alternata*.

#### 3.7.2. Bacteria

Bacterial growth and reproduction are major factors leading to the spoilage and reduced shelf life of agricultural products. The antibacterial effects of Q-GEL and RQ-GEL against *S. aureus* and *E. coli* are depicted in Figure 5a,c. The antibacterial efficacy of Q-GEL and RQ-GEL against *S. aureus* (94.13%, 93.79%) is stronger than that against *E. coli* (91.22%, 92.04%). This aligns with observations by Zhou et al., who reported similar antibacterial effects after crosslinking GTA with chitosan [10]. The antibacterial activity primarily results from the electrostatic interaction between cationic quaternary ammonium salts and negatively charged cell membranes or bacteria [10]. It is noteworthy that no significant differences were observed in the antibacterial activity of Q-GEL and RQ-GEL against *S. aureus* and *E. coli*. The results indicate that Q-GEL and RQ-GEL cling films exhibit excellent antibacterial activity, holding promising applications in the preservation of post-harvest agricultural products.

### 3.8. Determination of Antioxidant Activity

**Figure 6:**
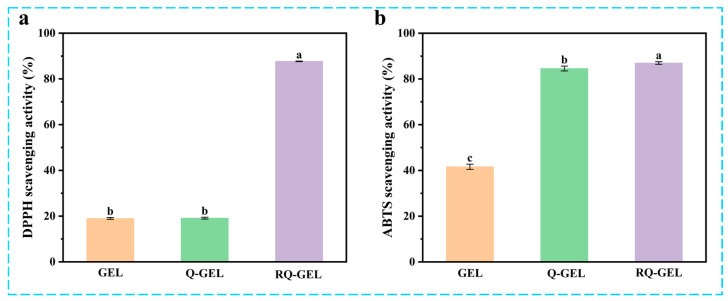
Effect of Q-GEL and RQ-GEL cling film on DPPH scavenging activity (**a**), ABTS scavenging activity (**b**). Vertical bars show the standard error of the means and different letters are significantly different at *p* < 0.05.

Post-harvest exposure of agricultural products to air can lead to oxidation, causing nutrient loss and diminishing the resistance of fruit peels [32]. Additionally, microbial invasion can induce further oxidative stress reactions, ultimately accelerating the aging of fruits and vegetables and shortening their shelf life [33]. Cling film materials with antioxidant activity can play a significant barrier role in protecting cherry tomatoes from free radical damage and maintaining their quality. As depicted in Figure 6a, the control group exhibits low DPPH radical scavenging activity, at only 18.99%. This is similar to the results of the antioxidant properties of gelatin itself (about 17%) measured by Boughriba et al. [23]. The antioxidant activity of Q-GEL film is measured at 19.10%, indicating that, after GTA modification, Q-GEL does not exhibit a significant improvement in antioxidant performance. However, with the addition of RosA, the RQ-GEL film shows a significant enhancement in free radical scavenging activity, reaching 87.69%. This is attributed to the four phenolic hydroxyl groups of RosA. Generally, the addition of phenolic substances to gelatin films increases their antioxidant performance [20,27].

In the ABTS experiment (Figure 6b), the control group exhibits an ABTS clearance capability of 41.6%, likely due to the presence of phenolic hydroxyl groups in aromatic amino acids within the gelatin matrix, such as tyrosine, resulting in its inherent free radical scavenging activity [23]. Notably, the ABTS clearance capability of the Q-GEL film reaches 84.6%, significantly higher than the control group. We posit that this is attributed to the -NH_4_^+^ groups in Q-GEL providing more hydrogen, facilitating substantial reduction in the ABTS^+^ free radical cation, thereby significantly enhancing its ABTS clearance capability [34]. The RQ-GEL film demonstrates an ABTS clearance capability of 87.0%, similar to the results obtained by Song et al. for epigallocatechin gallate/carvacrol-crosslinked gelatin films [22]. These findings indicate that the RQ-GEL film possesses robust antioxidant activity, making the prepared freshness film more suitable for preserving post-harvest agricultural products.

### 3.9. Preservation of Freshness Preservation 

#### 3.9.1. Preservation of Cherry Tomatoes

**Figure 7:**
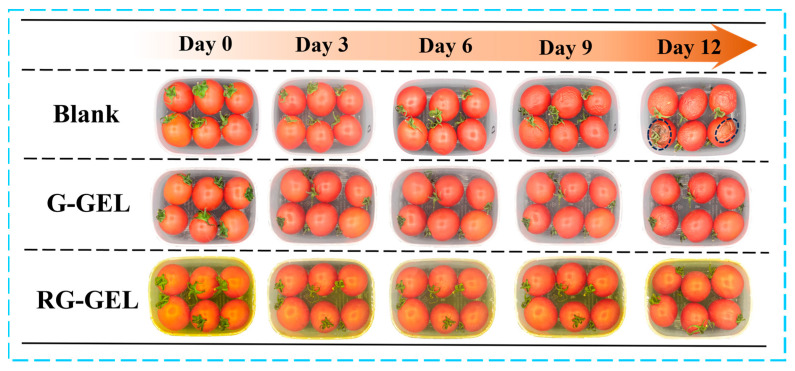
Photographs of the untreated cherry tomatoes and the Q-GEL and RQ-GEL covered cherry tomatoes during 12 days of storage.

During the storage of cherry tomatoes, electron photographs provide a visual record of their quality changes. To assess the potential application of Q-GEL and RQ-GEL cling films in preserving cherry tomatoes, a preservation study was conducted with a non-covered group as a control (Figure 7). In the early stages of storage, there were no significant differences in the visual quality between the control group and the Q-GEL and RQ-GEL groups. However, the control group exhibited noticeable concavity in some tomatoes, indicating a certain decline in quality by day 6. On day 9, all tomatoes in the control group showed extensive wrinkling, indicating further deterioration in quality. This is attributed to rapid moisture loss and oxidative metabolism, causing a decline in quality [35]. In contrast, the Q-GEL and RQ-GEL groups did not show significant visual changes. On day 12, cherry tomatoes in the control group displayed evident decay and extensive black spots. The proliferation of microorganisms resulted in complete quality degradation. In the Q-GEL group, some tomatoes showed wrinkling, indicating a decline in quality. However, the RQ-GEL group only exhibited mild wrinkling. Therefore, appropriate cling films can effectively delay the deterioration of cherry tomato quality.

#### 3.9.2. Weight Loss and Hardness

**Figure 8:**
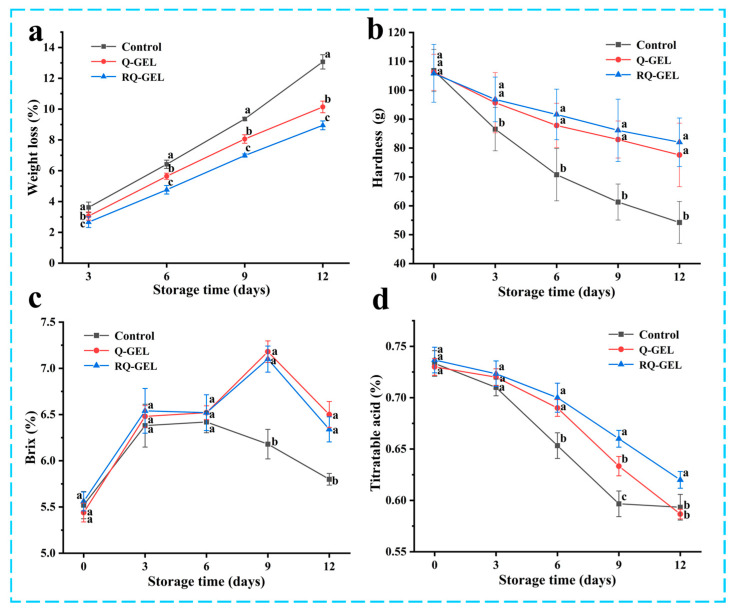
(**a**) Effect of Q-GEL and RQ-GEL cling film on weight loss (**a**), hardness (**b**), total soluble solids content (**c**), and titratable acid content (**d**). Vertical bars show the standard error of the means and different letters at the same storage time are significantly different at *p* < 0.05.

During 12 days of storage process, oxidative respiration, nutrient depletion by oxidative respiration and dehydration by transpiration are the main causes of weight loss in cherry tomatoes [6]. As shown in Figure 8a, all three groups exhibited weight loss during storage. The weight loss of cherry tomatoes in the control group was 13.07%. This may be attributed to the direct exposure of uncovered cherry tomatoes to the air, resulting in substantial internal moisture evaporation and microbial proliferation, further weakening the fruit’s natural immunity and exacerbating oxidative respiration. In contrast, the weight loss rates for the Q-GEL and RQ-GEL groups were 10.14% and 8.95%, respectively, significantly reducing the overall weight loss rate throughout the storage period and effectively maintaining the quality of cherry tomatoes.

As shown in Figure 8b, the hardness of all three groups of samples gradually decreased throughout the storage period, consistent with trends reported in previous studies [35]. Starting from the 3 days, the control group exhibited a sharp decline in hardness, significantly differing from the film-covered treatment groups. This decline is attributed to the respiratory and enzyme-related activities during storage, contributing to the degradation of cell structure [10] and resulting in reduced hardness. However, the presence of the cling film in the Q-GEL and RQ-GEL groups acted as a barrier to oxygen and carbon dioxide on both sides of the film, leading to a reduction in the respiratory rate of cherry tomatoes. On day 12, the hardness of the RQ-GEL group decreased only from 105.83 to 82.02 g, the Q-GEL group decreased from 106.20 to 77.61 g, while the control group’s hardness dropped from 106.82 to 54.23 g. These results indicate that the Q-GEL and RQ-GEL films effectively delay the decline in the hardness of cherry tomatoes.

#### 3.9.3. Total Soluble Solids and Titratable Acidity

The total soluble solids reflect the ripeness of the fruit and are closely associated with its flavor. As depicted in Figure 8c, the total soluble solids content in all three groups initially increased and then decreased. The initial metabolic processes involve the conversion of carbohydrates into sugars and other soluble compounds [36]. On day 9, the control group exhibited a declining trend in total soluble solids content, indicating that in the later stages of storage, oxidative respiration led to substantial nutrient depletion in tomatoes and microbial infestation, further accelerating nutrient loss. The Q-GEL and RQ-GEL groups also showed a decline in total soluble solids starting from day 12. These results indicate that film-covered treatments can preserve the freshness of cherry tomatoes and slow down the ripening process.

The titratable acidity, as shown in Figure 8d, exhibited a declining trend in all three sample groups throughout the storage period. From day 3 onwards, the control group experienced a rapid decrease in titratable acidity. The substantial depletion of total acids, attributed to the utilization of organic acids as substrates in the respiratory process, indicates an accelerated substance metabolism, leading to a rapid decline in tomato quality [6]. In contrast, the Q-GEL and RQ-GEL groups showed a gradual decline in total acid content. Significant differences between the RQ-GEL group and the control group emerged from the 6th day onwards. These results indicate that the RQ-GEL cling film can reduce the consumption of organic acids, thereby extending the shelf life of cherry tomatoes.

## 4. Conclusions

In this study, we developed an environmentally friendly cling film with antimicrobial and antioxidant properties for post-harvest agricultural products, using glycidyltrimethylammonium chloride and rosmarinic acid cross-linked gelatin as raw materials. The RQ-GEL film exhibited outstanding UV light barrier and mechanical properties. The FT-IR and XRD spectra indicated the formation of hydrogen bonds within RG-GEL, leading to a rearrangement of the internal structure and the formation of a denser mesh structure. The RQ-GEL film demonstrated significant inhibitory effects on fungi (*B. cinerea* and *A. alternata*.) and pathogenic bacteria (*S. aureus* and *E. coli*). Additionally, it exhibited a notable increase in DPPH and ABTS free radical scavenging activities. In the cherry tomato preservation experiment, compared to uncovered samples, the RQ-GEL film effectively reduced the weight loss rate, delayed the softening of the fruits, and prolonged the shelf life by mitigating the decline in soluble solids, titratable acidity, and total soluble solids content.

## Data Availability

Data are contained within the article and Supplementary Materials.

## Supplementary Materials

The following supporting information can be downloaded at: https://www.mdpi.com/article/10.3390/antiox13040431/s1. Figure S1. Crosslinking degree determination, absorbance values at 345 nm for background, control and experimental groups. Figure S2. Thicknesses of Q-GEL and RQ-GEL cling film.
