# Supplementary material for: Development and Characterization of Modified Gelatin-Based Cling Films with Antimicrobial and Antioxidant Activities and Their Application in the Preservation of Cherry Tomatoes
Source: Antioxidants (Basel). 2024 Apr 2;13(4):431. doi: 10.3390/antiox13040431 (PMC11047390; doi:10.3390/antiox13040431)
Supplement: Supplementary file 1 [file antioxidants-13-00431-s001.zip › antioxidants-2908429-supplementary.pdf]

Supporting Information

Development and characterization of modified gelatin-based cling films with antimicrobial and antioxidant activities and their application in the preservation of cherry tomatoes

Jianfu Qiao [a1] Linjing Wang [a1] Luxin Wang [a] Ziyan Li [a] Yue Huai [a] Shaoying Zhang [a*] Youwei Yu [a*]

a. College of Food Science, Shanxi Normal University, Taiyuan 030000, Shanxi, China

1. These authors contributed equally to this work and should be considered co-first authors.

* Corresponding author. **E-mail：** sxnuzsy@163.com (Shaoying Zhang)

E-mail： yywsxnu@163.com (Youwei Yu)

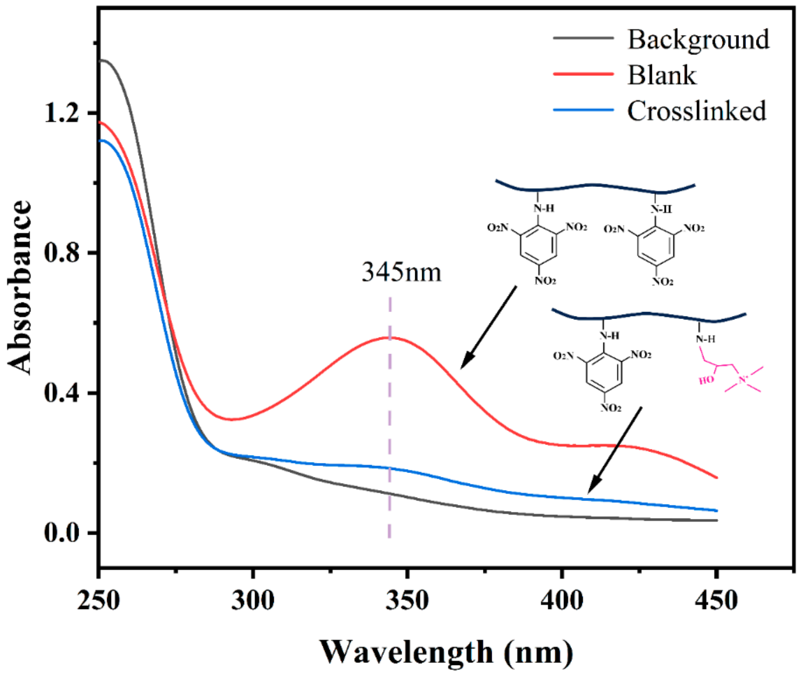

Figure S1. Crosslinking degree determination, absorbance values at 345 nm for background, control

and experimental groups.

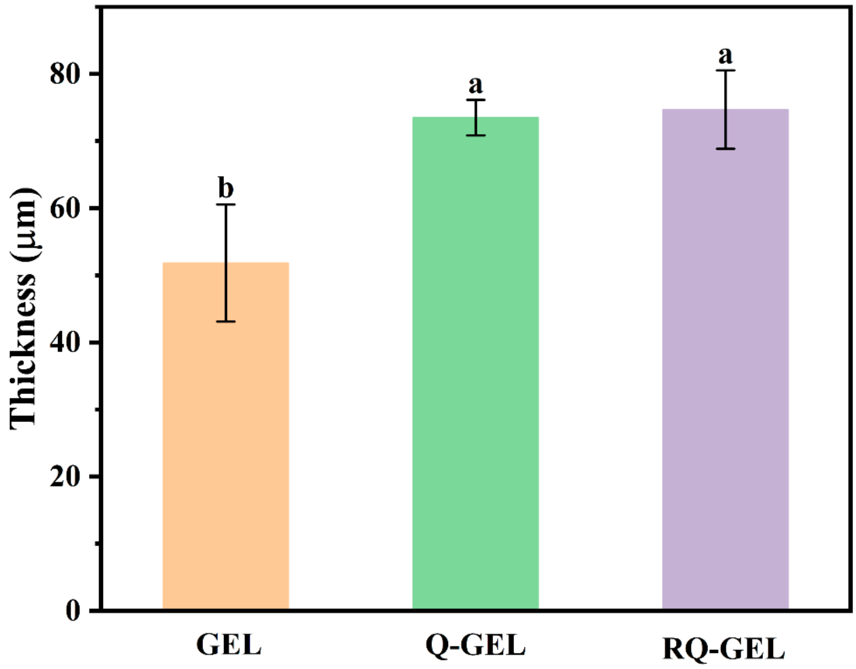

Figure S2. Thicknesses of Q-GEL and RQ-GEL cling film. Vertical bars show the standard error of the means and different letters are significantly different at $P < 0.05$.
